# Correlation of S100A4 and S100A14 Expression With Clinico-Pathological Features and Tumor Location in Colorectal Cancer Patients

**DOI:** 10.7759/cureus.65615

**Published:** 2024-07-29

**Authors:** Sardar H Arif

**Affiliations:** 1 Surgery, College of Medicine, University of Duhok, Duhok, IRQ

**Keywords:** clinico-pathological features, tumor location, s100a14, s100a4, colorectal cancer

## Abstract

Background

Colorectal cancer (CRC) remains a major cause of morbidity and mortality worldwide. Understanding the clinical and pathological characteristics of CRC patients is essential for improving diagnosis, treatment, and prognostication. S100 proteins play a crucial role in CRC by promoting tumor growth, metastasis, and inflammation through their involvement in various cellular processes such as proliferation, migration, and immune response modulation. Elevated levels of specific S100 proteins have been associated with poor prognosis and serve as potential biomarkers for early detection and therapeutic targets in CRC. This study aims to analyze the general and medical characteristics of CRC patients, with a particular focus on the expression patterns of S100A4 and S100A14 proteins and their correlation with tumor location and various clinical parameters.

Methods

This cross-sectional study included 98 CRC patients aged 21 to 92 years. Clinical data were collected from Vajeen Hospital (Duhok/ Iraq), including age, gender, and presenting symptoms. Pathological data such as tumor site, tumor size, tumor, node, and metastasis (TNM) stage, tumor grade angio-lymphatic invasion, perineural invasion, and metastasis were analyzed. The expression of S100A4 and S100A14 proteins was assessed using immunohistochemistry, and their correlation with clinico-pathological features and tumor location was evaluated using statistical analysis.

Results

The 98 patients with a mean age of 57.27 years. The majority were over 50 years old (68, 69.39%) with a nearly equal gender distribution. The most common symptom was bleeding per rectum (36, 36.74%). TNM staging revealed 25.51% (n=25) of patients at stage I, 32.65% (n=32) at stage II, 24.49% (n=24) at stage III, and 17.35% (n=17) at stage IV. Angio-lymphatic invasion was present in 65.31% (n=64) of patients, and lymph node invasion in 38.78% (n=38). All tumors were adenocarcinomas, with 82.65% (n=81) being intermediate grade. S100A4 expression was low in early-stage tumors but significantly higher in advanced stages (P < 0.0001). High S100A4 expression was associated with vascular invasion (P = 0.0006), perineural invasion (P = 0.0002), lymph node invasion (P < 0.0001), and metastasis (P = 0.0010). S100A14 expression was inversely correlated with disease severity. Low S100A14 expression was more common in advanced stages (P < 0.0001) and was associated with higher rates of vascular invasion (P = 0.0018), lymph node invasion (P < 0.0001), and metastasis (P = 0.0001).

Conclusion

This study highlights significant correlations between S100A4 and S100A14 expression with various clinico-pathological features in CRC patients. High S100A4 expression is linked with tumor aggressiveness, whereas low S100A14 expression is associated with advanced disease stages and increased metastasis. However, there is no observed correlation between the expression of these proteins and the tumor site.

## Introduction

Colorectal cancer (CRC) remains a significant global health challenge, being the third most commonly diagnosed cancer and the second leading cause of cancer-related deaths [1]. Despite advances in screening and treatment, the prognosis for patients with advanced CRC remains poor, highlighting the urgent need for a deeper understanding of the molecular mechanisms driving its progression. Among the various molecular players involved, the S100 protein family has garnered considerable attention due to its role in cancer development and metastasis. In particular, S100A4 and S100A14 have emerged as pivotal regulators in the context of CRC [2].

S100A4, also known as metastasin, is well-documented for its role in promoting metastasis in various cancers, including CRC. This calcium-binding protein facilitates tumor progression by enhancing cell motility, invasion, and angiogenesis, ultimately contributing to the metastatic spread. Elevated levels of S100A4 have been correlated with poor prognosis and increased tumor aggressiveness, making it a potential biomarker for disease progression and a target for therapeutic intervention [3].

On the other hand, S100A14, although less studied than S100A4, is increasingly recognized for its dual role in tumorigenesis. Intriguingly, S100A14 appears to exhibit both tumor-suppressive and tumor-promoting functions depending on the cellular context and cancer type [4]. In CRC, emerging evidence suggests that S100A14 might influence cancer progression through its interactions with various signaling pathways, impacting cellular proliferation, apoptosis, and migration [5]. However, the exact mechanisms by which S100A14 modulates CRC progression remain to be fully elucidated.

The anatomical site of the tumor within the colon or rectum is another critical factor influencing CRC behavior and patient outcomes. Right-sided (proximal) and left-sided (distal) CRCs are known to exhibit distinct molecular characteristics and clinical manifestations [6]. This anatomical distinction may also play a role in modulating the expression and function of S100A4 and S100A14, thereby affecting tumor behavior and progression differently depending on the site of origin.

This article aims to explore the complex interplay between S100A4 and S100A14 in CRC progression, with a particular focus on how their expression and function may vary according to the anatomical location of the tumor.

## Materials and methods

Tissue specimens and patients

After obtaining informed consent and securing approval from the Research Ethics Committee of the College of Medicine at the University of Duhok, a study was conducted with 98 patients diagnosed with CRC. Participants were over 20 years of age, had a confirmed histopathological diagnosis of CRC, and were scheduled to undergo surgery, chemotherapy, or radiotherapy. Histopathological samples from these patients were subjected to immunohistochemical analysis. Patients were excluded if they declined to participate, had incomplete examination data (including demographics, CRC location, staging, and histopathological characteristics), had unreadable immunohistochemical results, or had autoimmune and inflammatory diseases. Following surgical resection, tissue specimens were fixed in 10% neutral buffered formalin and then embedded in paraffin blocks. Sections of tissue, each four micrometers thick, were stained with hematoxylin and eosin (H&E) for detailed histopathological examination.

Immuhistochemistry (IHC)

Deparaffinized tissue slides were treated with mouse monoclonal primary antibodies against S100A4 and S100A14 proteins (Santa Cruz, USA). A secondary detection system (DAKO, Denmark) with a conjugated polymer was used to bind to the primary antibody. For permanent color development and microscopic detection, DAB chromogen was utilized. Tissue slides were heated on a hot plate at 60°C for 30 minutes, then immersed in two changes of xylene, followed by three different concentrations of ethanol (100%, 95%, and 80%). The slides were rinsed with distilled water to remove ethanol residue. Next, the slides were placed in EDTA buffer pH 9.0 (DAKO) for target retrieval and heated in a microwave. After cooling to room temperature, they were rinsed with Tris Buffered Saline (TBS) with Tween 20. The slides were then covered with a peroxidase-blocking solution (DAKO) and rinsed again with TBS with Tween 20. Following this, 200 μL of the primary antibody (S100A4 at 1:200 and S100A14 at 1:100) was applied to the tissue slides, followed by another rinse with TBS with Tween 20. Two drops of the mouse secondary antibody (DAKO) were added, and the slides were rinsed once more with TBS with Tween 20. Subsequently, DAB substrate (DAKO) was applied, and the slides were rinsed, immersed in hematoxylin, and passed through four different concentrations of ethanol (100%, 95%, and 80%). Finally, the slides were immersed in two changes of xylene, and then the cover slipped.

Immunohistochemistry scoring

The expression of S100A14 was predominantly observed on the cell membrane, whereas S100A4 expression was localized to the nucleus and cytoplasm. Two independent pathologists evaluated protein expression using a semi-quantitative scoring system that accounted for both staining intensity and the proportion of positive cells. The intensity of staining was classified as follows: negative (0), weak (1), moderate (2), and strong (3). The percentage of cells showing positive staining was categorized into five groups: 0%-5% (0), 6%-25% (1), 26%-50% (2), 51%-75% (3), and 76%-100% (4). A staining index score, ranging from 0 to 12, was derived by multiplying the intensity score by the proportion score. For statistical purposes, a final score of 0-6 indicated low protein expression, while a score of 7-12 indicated high expression [7].

Statistical analyses

The general and medical characteristic of patients with CRC were presented in mean and standard deviation or number and percentage. The tumor-related characteristics of patients with CRC were presented in number and percentage. The association of S100A4 and S100A14 with medical characteristics of patients with CRC were examined in Pearson chi-squared tests (Chi-squared or Fishers’ exact test as appropriate). The magnitude of association was determined in in Odds Ratio and the uncertainty of the association in 95% confidence interval (CI). The statistical calculations were performed using JMP®, Version 17.1.0 (SAS Institute Inc., Cary, NC, 1989-2020) (https://www.jmp.com/en_us/home.html).

Ethical approval

The ethical approval of this study was obtained from the local health ethics committee in Duhok General Directorate of Health registered on May 29, 2024 as reference number 29052024-4-15. No intervention was applied on the patients in this study.

## Results

General and medical characteristics of CRC patients

The clinical presentation of CRC was analyzed in 98 patients, with ages ranging from 21 to 92 years (mean age: 57.27 years, standard error mean: 1.50). The majority of the patients were over 50 years old (n=68), comprising 69.39% of the samples, while 30.61% (n=30) were between 18 and 50 years old. The gender distribution was nearly equal, with males making up 52.04% (n=51) and females 47.96% (n=47). Regarding the clinical symptoms at presentation, the most common was bleeding per rectum, observed in 36.74% (n=36) of the patients. Intestinal obstruction was reported by 17.35% (n=17), and abdominal pain by 16.33% (n=16). Anemia and constipation each accounted for 8.16% (n=8) of the cases. Bloody diarrhea was noted in 6.12% (n=6) of patients, whereas loss of weight, melena, and the presence of a right iliac fossa (RIF) mass each occurred in 2.04% (n=2) of patients. A small number of patients, 1.02%, presented with perforation. These findings highlight the varied clinical manifestations of CRC, emphasizing the importance of recognizing a wide range of symptoms for early diagnosis and management (Table 1).

**Table 1:** General and medical characteristics of patients with colorectal cancer RIF - Right Iliac fossa

General characteristics (n=98)	Number	Percent (%)
Age (Range: 21-92 years) Std Err Mean: 1.50	57.27	14.89
Age groups		
18-50	30	30.61
> 50 years	68	69.39
Gender		
Male	51	52.04
Female	47	47.96
Presentation		
Bleeding per rectum	36	36.74
Intestinal obstruction	17	17.35
Abdominal pain	16	16.33
Anemia	8	8.16
Constipation	8	8.16
Bloody diarrhea	6	6.12
Loss of weight	2	2.04
Melena	2	2.04
RIF mass	2	2.04
Perforation	1	1.02

Clinico-pathological characteristics of CRC patients

The study consisted of 98 CRC patients with diverse medical characteristics. The TNM staging revealed that 25.51% (n=25) of patients were at stage I, 32.65% (n=32) at stage II, 24.49% (n=24) at stage III, and 17.35% (n=17) at stage IV, indicating a relatively even distribution with a slight predominance in early stages. Angio-lymphatic invasion was present in 65.31% of patients, suggesting a higher prevalence of this aggressive feature. Perineural invasion was observed in 38.78% (n=38) of the cohort, while lymph node invasion was also noted in 38.78% (n=38) of patients. Tumor grading showed that 82.65% (n=81) of tumors were intermediate grade, 11.22% (n=11) were high grade, and 6.12% (n=6) were low grade. Regarding tumor location, 36.74% (n=36) were in the right colon, 33.67% (n=33) in the left colon, 26.53% (n=26) in the rectum, and 3.06% (n=3) in the transverse colon. Tumor size analysis indicated that 36.74% of tumors (n=33) were smaller than 5 cm, whereas 63.26% (n=62) were 5 cm or larger. Metastasis was found in 21.43% (n=21) of patients. Notably, all tumors were histologically classified as adenocarcinoma, underscoring the uniformity of tumor type within this study (Table 2).

**Table 2:** Clinico-pathological characteristics of patients with colorectal cancer TNM - tumor, node, and metastasis

Medical characteristics (n=98)	Number	Percent (%)
TNM stage		
I	25	25.51
II	32	32.65
III	24	24.49
IV	17	17.35
Angio-lymphatic invasion		
No	34	34.69
Yes	64	65.31
Perineural invasion		
No	60	61.22
Yes	38	38.78
Lymphnode invasion		
No	60	61.22
Yes	38	38.78
Tumor grade		
Low	6	6.12
Intermediate	81	82.65
High	11	11.22
Tumor site		
Right colon	36	36.74
Left colon	33	33.67
Rectum	26	26.53
Transverse colon	3	3.06
Tumor size		
<5 cm	36	36.74
≥ 5 cm	62	63.26
Metastasis		
No	77	78.57
Yes	21	21.43
histologic type adenocarcinoma	98	100

Correlation of S100A4 expression with clinico-pathological features and CRC tumor location

**Figure 1:**
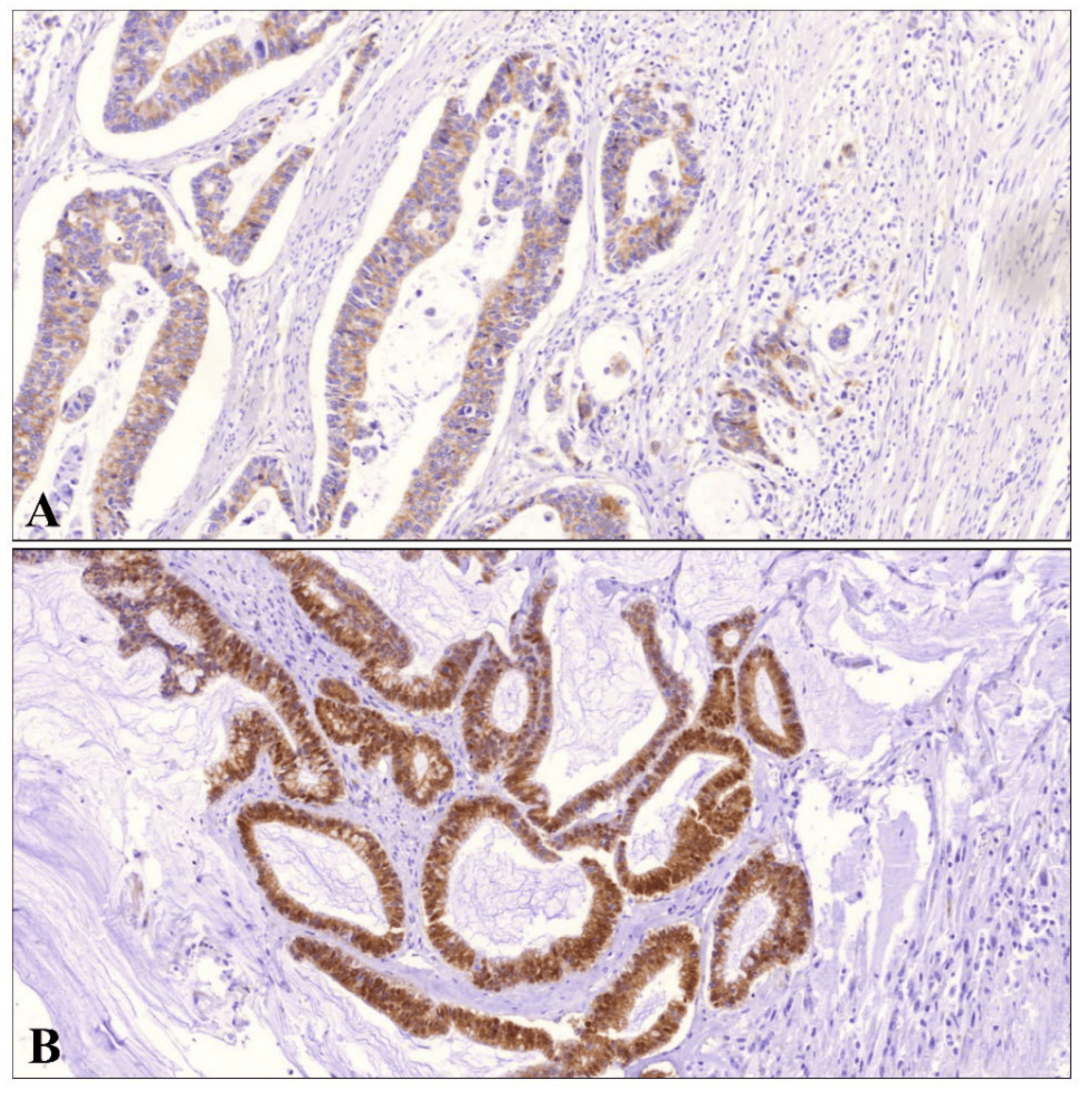
Expression of S100A4 in CRC tissues. S100A4 protein were found to be expressed mainly on the cytoplasm and nucleus of colon cancer cells. (A) Faint signals from this protein was detected in early stages of CRC. (B) Whereas S100A4 was strongly expressed in the advanced stage of CRC (20x). CRC - colorectal cancer

The analysis of S100A4 expression in relation to various medical characteristics in 98 CRC patients revealed that S100A4 expression was localized to the nucleus and cytoplasm and its expression significant associations with several clinical parameters (Figures 1A, 1B, Table 3). Low S100A4 expression was predominantly observed in early-stage tumors, with 50.00% of stage I patients exhibiting low levels compared to only 3.85% showing high levels. Conversely, high S100A4 expression was significantly more prevalent in advanced stages, seen in 40.38% (n=21) of stage III and 30.77% (n=16) of stage IV patients (P < 0.0001). Vascular invasion was significantly associated with high S100A4 expression, with 80.77% of patients (n=42) with vascular invasion showing high levels, compared to 19.23% (n=10) with low levels (P = 0.0006). Perineural invasion also correlated with high S100A4 expression, present in 55.77% (n=29) of high-expression cases compared to 19.57% (n=9) in low-expression cases (P = 0.0002). Lymph node invasion exhibited a striking difference, with high S100A4 expression found in 65.38% (n=34) of affected patients, while only 8.70% (n=4) of those without lymph node invasion showed high levels (P < 0.0001). Tumor grade analysis indicated a trend towards higher S100A4 expression with increasing grade, although this was not statistically significant (P = 0.1278). High expression was seen in 76.92% (n=40) of intermediate grade and 17.31% (n=9) of high-grade tumors. Tumor site did not significantly affect S100A4 expression, with similar distributions across right colon (18, 34.62%), left colon (20, 38.46%), rectum (13, 25.00%), and transverse colon (one, 1.92%) (P = 0.6924). Tumor size was also not significantly associated, though high S100A4 expression tended to be more common in larger tumors (≥5 cm) at 69.23% (n=36), compared to 30.77% (n=16) in smaller tumors (<5 cm) (P = 0.1928). Finally, metastasis showed a strong correlation with high S100A4 expression, observed in 34.62% (n=18) of metastatic cases versus 6.52% (n=3) of non-metastatic cases (P = 0.0010). These findings underscore the potential role of S100A4 as a marker of tumor aggressiveness and progression in CRC.

**Table 3:** Association between S100A4 expression and clinico-pathological features of CRC patients CRC - colorectal cancer

Clinico-pathological features	S100A4 outcome, no (%) by column			P-value
	Low expression (n=46)	High expression (n=52)	OR (95% CI)	
TNM stage				
I	23 (50.00)	2 (3.85)	Reference	<0.0001
II	19 (41.30)	13 (25.00)	7.87 (1.58-39.28)	
III	3 (6.52)	21 (40.38)	80.5 (12.23-529.91)	
IV	1 (2.17)	16 (30.77)	184 (15.35-2205.57)	
Vascular invasion				
No	24 (52.17)	10 (19.23)	Reference	0.0006
Yes	22 (47.83)	42 (80.77)	4.58 (1.86-11.27)	
Perineural invasion				
No	37 (80.43)	23 (44.23)	Reference	0.0002
Yes	9 (19.57)	29 (55.77)	5.18 (2.08-12.89)	
Lymph node invasion				<0.0001
No	42 (91.30)	18 (34.62)	Reference	
Yes	4 (8.70)	34 (65.38)	0.05 (0.02-0.16)	
Tumor grade				
I	3 (6.52)	3 (5.77)	Reference	0.1278
II	41 (89.13)	40 (76.92)	0.98 (0.19-5.12)	
III	2 (4.35)	9 (17.31)	4.5 (0.49-41.25)	
Tumor site				
Right colon	18 (39.13)	18 (34.62)	Reference	0.6924
Left colon	13 (28.26)	20 (38.46)	1.54 (0.59-4.0)	
Rectum	13 (28.26)	13 (25.00)	1 (0.36-2.74)	
Transverse colon	2 (4.35)	1 (1.92)	0.5 (0.04-6.02)	
Tumor size				
<5 cm	20 (43.48)	16 (30.77)	Reference	0.1928
≥ 5 cm	26 (56.52)	36 (69.23)	1.73 (0.76-3.94)	
Metastasis				
Negative	43 (93.48)	34 (65.38)	Reference	0.0010
Positive	3 (6.52)	18 (34.62)	7.59 (2.06-27.91)	

Correlation of S100A14 expression with clinico-pathological features and CRC tumor location

**Figure 2:**
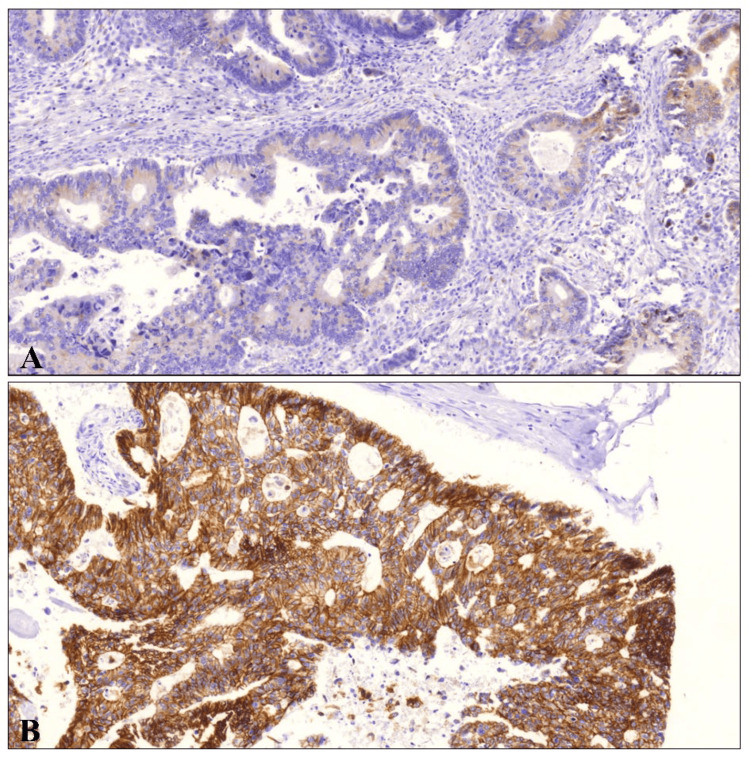
Expression of S100A14 in CRC tissues. (A) Low expression of S100A14 was found along the cell membrane of advanced stage of CRC. (B) Contrary, strong expression of this protein was detected in early stage of CRC (20x). CRC - colorectal cancer

The S100A14 proteins were predominantly expressed on the membranes of colon cancer cells and its expression significantly correlated with several parameters (Figures 2A, 2B, Table 4). When considering TNM stage, low S100A14 expression was more prevalent in advanced stages, with 36.84% (n=14) of low expression cases in stage IV, compared to just 5.00% (n=3) in high expression cases (P < 0.0001). Vascular invasion was notably higher in low S100A14 expression cases, at 84.21% (n=32), compared to 53.33% (n=32) in high expression cases (P = 0.0018). Perineural invasion, however, did not show a significant difference (P = 0.3351). Lymph node invasion was significantly more common in low S100A14 expression cases (29, 76.32%) compared to high expression cases (nine, 15.00%), with a highly significant P-value (<0.0001). Tumor grade distribution did not significantly differ between the groups (P = 0.1979). However, tumor site analysis revealed some differences, with rectal tumors being more common in high S100A14 expression cases (35.00%) compared to low expression cases (five, 13.16%), albeit with a P-value of 0.0857. Tumor size did not differ significantly between the groups (P = 0.9860), nor did the presence of tumors in the right or left colon and transverse colon. Metastasis was significantly less common in high S100A14 expression cases (five, 8.33%) compared to low expression cases (16, 42.11%), with a P-value of 0.0001. Overall, the study highlights that low S100A14 expression is associated with more advanced disease stages, higher vascular and lymph node invasion, and a higher incidence of metastasis, indicating a potentially important role of S100A14 in cancer progression.

**Table 4:** Association between S100A14 expression and clinico-pathological features of CRC patients CRC - colorectal cancer

Clinico-pathological features	S100A14 outcome, no (%) by column			P-value
	Low expression (n=38)	High expression (n=60)	OR (95% CI)	
TNM stage				
I	4 (10.53)	21 (35.00)	Reference	<0.0001
II	4 (10.53)	28 (46.67)	1.33 (0.3-5.96)	
III	16 (42.11)	8 (13.33)	0.1 (0.02-0.37)	
IV	14 (36.84)	3 (5.00)	0.04 (0.01-0.21)	
Vascular invasion				
No	6 (15.79)	28 (46.67)	Reference	0.0018
Yes	32 (84.21)	32 (53.33)	0.21 (0.08-0.59)	
Perineural invasion				
No	21 (55.26)	39 (65.00)	Reference	0.3351
Yes	17 (44.74)	21 (35.00)	0.67 (0.29-1.53)	
Lymphnode invasion				
No	9 (23.68)	51 (85.00)	Reference	<0.0001
Yes	29 (76.32)	9 (15.00)	0.05 (0.02-0.15)	
Tumor grade				
I	2 (5.26)	4 (6.67)	Reference	0.1979
II	29 (76.32)	52 (86.67)	0.9 (0.15-5.2)	
III	7 (18.42)	4 (6.67)	0.29 (0.04-2.32)	
Tumor site				
Right colon	15 (39.47)	21 (35.00)	Reference	0.0857
Left colon	17 (44.74)	16 (26.67)	0.67 (0.26-1.74)	
Rectum	5 (13.16)	21 (35.00)	3.0 (0.92-9.75)	
Transverse colon	1 (2.63)	2 (3.33)	1.43 (0.12-17.23)	
Tumor size				
<5 cm	14 (36.84)	22 (36.67)	Reference	0.9860
≥ 5 cm	24 (63.16)	38 (63.33)	1.01 (0.43-2.34)	
Metastasis				
Negative	22 (57.89)	55 (91.67)	Reference	0.0001
Positive	16 (42.11)	5 (8.33)	0.13 (0.04-0.38)	

## Discussion

The S100 protein family plays a crucial role in various intracellular and extracellular activities, including modulation of cell differentiation and proliferation, cell-cell communication, intracellular signaling, cell motility, and cell structure [8]. Several S100 proteins have been implicated in promoting malignancy, with altered expression in tumors serving as important prognostic and diagnostic indicators [9]. S100A4, in particular, has been identified as a significant immunohistochemical marker in CRC, offering additional prognostic information. Over-expression of S100A4 has been reported in multiple cancers, such as gastric [10,11], colorectal and breast cancers [12,13]. Recent studies suggest that the nuclear localization of S100A4 is associated with tumor stage in CRC and may play a role in gene regulatory pathways related to metastatic phenotypes [14].

In the current study, the correlation between S100A4 expression and the age and gender groups were not significant, these results were compatible with studies done by Wenlong et al. and Jung et al. [15,16]. Regarding the site of the tumor and expression of this protein, results showed a non-significant correlation between these variables in the present study, while in a study done by Kazakova et al., S100A4 expression was more in the rectum than the other parts [17].

In the present study, S100A4 over-expression was observed in 52 (53.06%) of the 98 colorectal adenocarcinoma tissue specimens, correlating closely with factors indicative of tumor aggressiveness, such as lymph node metastasis, depth of invasion, and peritoneal dissemination. This finding aligns with other research indicating a higher frequency of S100A4 over-expression in cancer cells compared to normal colonic mucosa. These results corroborate the broader body of work linking S100A4 expression with increased tumor aggressiveness [18,19]. 

S100A14, a newer member of the S100 protein family, shows differential regulation in tumors and is highly expressed in several epithelial tissues [20], particularly in the colon [21]. Various studies have reported different expression patterns of S100A14 in tumors [22-24], often noting genomic instability in the chromosomal region 1q21 where S100A14 is located, alongside related genes such as S100A16, S100A13, and S100A1 [25]. This study reveals a strong inverse relationship between S100A14 expression and several key pathological features in CRC, underscoring its potential as a prognostic marker.

In the present study, the correlation between the age and the gender groups and S100A14 expression was not significant, these results were similar to study done by Hashida and Coffey [26]. Regarding to the correlation between the site of tumor and expression of this protein results showed non-significant relation between them, while in study done by Diamantopoulou et al., S100A14 expression was more in the right side of the colon than the other parts [25].

Notably, low S100A14 expression was significantly more prevalent in advanced TNM stages, particularly stage IV, suggesting that reduced S100A14 expression may be associated with tumor progression and metastasis. Supporting this, Wang et al. found lower S100A14 levels in malignant tissues compared to normal tissues, with reduced expression in poorly differentiated tumors and cases with distant metastases. However, they did not observe significant differences in terms of depth of infiltration (T) and lymph node metastases (N). Additionally, they reported worse survival outcomes in patients lacking S100A14 expression [26]. These findings collectively highlight the importance of S100A14 as a marker for CRC prognosis and its potential role in tumor biology.

Limitations

This study has several limitations that need to be acknowledged. First, the sample size of 98 patients, while sufficient to observe significant associations, may not capture the full variability of CRC presentations and molecular characteristics. Second, the study is limited to a single geographical region, which may affect the generalizability of the findings to other populations. Third, the cross-sectional design of the study precludes establishing causal relationships between protein expression and disease progression. Lastly, while immunohistochemistry and next-generation sequencing provide robust data, the study would benefit from complementary techniques such as Western blotting or RT-PCR to validate the expression levels of S100A4 and S100A14.

## Conclusions

In conclusion, the study highlights the significant correlation between S100A4 and S100A14 expression with various clinical and pathological features in CRC patients. Low S100A14 expression is associated with more advanced disease stages, higher rates of vascular and lymph node invasion, and a higher incidence of metastasis. This underscores the potential of S100A14 as a valuable prognostic marker in CRC. Additionally, the over-expression of S100A4, observed in over half of the tumor samples, further supports its role in tumor aggressiveness and progression. However, the expression of these S100 proteins does not correlate with the tumor site and shows no statistically significant association. These findings contribute to a better understanding of the molecular underpinnings of CRC and emphasize the need for incorporating these markers in clinical practice to enhance patient stratification and management.
